# Equity considerations for the implementation of health insurance benefit package in Ethiopia: result of expert Delphi exercise

**DOI:** 10.1186/s12939-024-02226-z

**Published:** 2024-09-11

**Authors:** Solomon Tessema Memirie, Muluken Argaw, Mieraf Taddesse Tolla, Frehiwot Abebe, Wubaye Walelgne Dagnaw, Ole F. Norheim, Amanuel Yigezu

**Affiliations:** 1 Addis Center for Ethics and Priority Setting, College of Health Sciences, Addis Ababa University, Addis Ababa, Ethiopia; 2 Bergen Centre for Ethics and Priority Setting, Department of Global Public Health and Primary Care, University of Bergen, Bergen, Norway; 3 Ethiopian Health Insurance Service, Addis Ababa, Ethiopia; 4 Center for Integration Science, Department of Global Health Equity, Brigham and Women’s Hospital, Boston, MA USA; 5 Trinity College Institute for Neuroscience, Trinity College Dublin, Dublin, Ireland; 6 School of Medicine, Trinity College Dublin, Dublin, Ireland

**Keywords:** Equity, Health benefit package, Cost-effectiveness analysis, Universal health coverage, Ethiopia

## Abstract

**Background:**

Efficiency, equity and financial risk protection are key health systems objectives. Equitable distribution of health care is among the priority strategic initiative of the government of Ethiopia. However, data on the distribution of interventions benefits or on disease burden disaggregated by subpopulations to guide health care priority setting is not available in Ethiopia.

**Methods:**

Aligned with policy documents, we identified the following groups to be the worse off in the Ethiopian context: under-five children, women of reproductive age, the poor, and rural residents. We used the Delphi technique by a panel of 28 experts to assign a score for 253 diseases/conditions over a period of two days, in phases. The expert panel represented different institutes and professional mix. Experts assigned a score 1 to 4; where 4 indicates disease/condition predominantly affecting the poor and rural residents and 1 indicates a condition more prevalent among the wealthy and urban residents. Subsequently, the average equity score was computed for each disease/condition.

**Results:**

The average scores ranged from 1.11 (for vitiligo) to 3.79 (for obstetric fistula). We standardized the scores to be bounded between 1 and 2; 1 the lowest equity score and 2 the highest equity score. The scores for each disease/condition were then assigned to their corresponding interventions. We used these equity scores to adjust the CEA values for each of the interventions. To adjust the CEA values for equity, we multiplied the health benefits (the denominator of the cost-effectiveness value) of each intervention by the corresponding equity scores, resulting in equity adjusted CEA values. The equity adjusted CEA was then used to rank the interventions using a league table.

**Conclusions:**

The Delphi method can be useful in generating equity scores for prioritizing health interventions where disaggregated data on the distribution of diseases or access to interventions by subpopulation groups are not available.

**Supplementary Information:**

The online version contains supplementary material available at 10.1186/s12939-024-02226-z.

## Introduction

Prioritizing a pro poor pathway to achieve universal health coverage (UHC) is considered a key component of development [1]. UHC entails that high quality essential services are provided to everyone without the consequences of financial hardship. A country’s ability to finance health is closely correlated with its wealth; poorer countries spend on health much less than wealthier countries [2]. No country, however, has sufficient resources to meet all the aspirations of UHC, where the costs of new technologies and medicines are rising exponentially. In the face of resource constraints, individual countries are required to determine their own definition of “essential”. UHC cannot provide access to all available health services. However, it should consider a comprehensive list of top priority services that also addresses other social goals such as equity and financial risk protection [3]. These require countries to make trade-offs, make choices and set priorities.

In the quest for UHC, many low- and low-middle income countries have designed or redesigned their essential package of health services (EPHS) [4]. Ethiopia is one of the countries, which in 2019 revised its essential health services package (EHSP) [5]. The development of Ethiopia’s EHSP employed the following prioritization criteria: disease burden, cost effectiveness, equity, financial risk protection, budget impact, public acceptability and political acceptability and included 1018 interventions in its final list [5]. However, subsequent assessment focusing on the status of health services delivered, health financing and the health workforce were found to be major challenges in EHSP implementation in Ethiopia [6]. Ethiopia has limited fiscal space for public health expenditure (government spending for health per capita of US$6) which is substantially lower than what is required to implement the package (with an estimated ratio between the EHSP cost per capita in the first year of implementation and government spending for health of 6.7) [6].

The government of Ethiopia in 2010 adopted the community based (CBHI) and social health insurance (SHI) systems with the aim reducing financial barrier in access to services and offer FRP [7]. In the current CBHI system, the health benefits package is negatively listed and the package is not evaluated by the interventions cost-effectiveness and affordability, which may result in implicit rationing at facility level^1^. To address these challenges, the Ethiopian Health Insurance Service (EHIS) aimed to define an explicit health insurance benefit package (HIBP).

The HIBP development used a multicriteria decision analysis (MCDA) through a transparent and participatory approach. The key criteria for the MCDA were cost-effectiveness, equity and financial risk protection of interventions for the prevailing disease conditions in Ethiopia, aligned with recommendations by the World Health Organizations (WHO) [3]. Economic evaluation evidence was sought from literature review locally and other comparable settings where cost-effectiveness ratio (CER) of relevant interventions were extracted and presented in a league table. Details of economic evaluation and financial risk protection are discussed separately in upcoming papers. To evaluate equity, the following attributes were considered: economic status, area of residence, gender and age. There is substantial variation in access to health care services and health outcomes by socioeconomic status, area of residence and age in Ethiopia [8–10]. Therefore, in the development of the HIBP, interventions that are more likely to benefit the socioeconomically marginalized groups carry more weight and would have a larger equity impact. However, disaggregated data by socioeconomic status and area of residence for most of the interventions or their corresponding conditions/diseases is not available in Ethiopia. Therefore, we used a Delphi method (using panel of experts) to assign an equity score for each disease condition and subsequently their corresponding interventions based on which segment of the population (poor vs. rich, rural vs. urban, etc.) is more likely to be affected by the specific disease/condition. Below, we present how the equity scores were generated, the result of the exercise and subsequent use of the equity scores in prioritizing health interventions (along with other criteria such as cost-effectiveness ratio) to populate the HIBP in Ethiopia.

## Methods

### Development of equity attributes

Health is a human right. Everyone, regardless of sex, area of residence, age, socioeconomic status, is entitled to health services, medicines and equipment that are available, accessible, acceptable and of good quality [11]. Enhancing the provision of equitable and quality comprehensive health services delivery is a core strategic direction of the second health sector transformation plan (HSTP-II) in Ethiopia [12]. In accordance with the HSTP II and national health equity strategic plan, the core team identified four equity dimensions: socioeconomic status/conditions, gender, geography and age; with the aim of putting extra weight to interventions that address the health care needs of vulnerable, marginalized, and disadvantaged groups [8–10, 12, 13]. Since income and area of residence (urban/rural) are the main predictors of differences in health services utilization and health outcome, we aimed to assess these attributes in our exercise [12, 14].

After identifying the attributes, the core team decided to use an expert panel to assign equity scores to disease conditions rather than interventions since different disease conditions affect different segments of the population while access to interventions show a pro-rich distribution [15]. Based on the Global Burden of Diseases (GBD) listing for Ethiopia, the core team identified 253 disease conditions/groups (that address the prevailing disease conditions in Ethiopia) for equity scoring and developed a questionnaire to guide the expert panel (details of the questionnaire are included in additional file I) [16]. Each disease condition/group were scored on a scale of 1 to 4. For both income and area of residence, we used a dichotomous approach (urban vs. rural; poor vs. rich). The highest rating (a score of 4) was assigned to conditions that are likely more prevalent among the poor and rural residents while a score of 1 represent diseases that do not specifically affect the worst off (likely equally distributed among the worse off and the better off or more prevalent among the better off). A single score was assigned to both attributes (income and area of residence) considered together.

### Details of expert panel

Twenty-eight experts participated in the workshop; 29% were females. We selected participants for the workshop from all tiers of the health system (health center, primary hospital, general and tertiary hospitals), from different regions in Ethiopia. In addition, patient associations, representatives from non-governmental organizations, Federal Ministry of Health, Universities, and public health associations were part of the expert panel. The professional mix included: clinicians (different specialist clinicians, family medicine, general practitioners), researchers, public health experts, reproductive health experts, epidemiologist, nurses, health managers and patient group leaders (details of the participants list is included in additional file III).

### The Delphi exercise

We divided the scoring exercise into two sessions. In the first session, we described the purpose of the exercise, the prevailing disease burden in Ethiopia and disaggregated disease burden data when available. We presented disaggregated disease burden data by wealth and geographic location for a few conditions where such evidence was available from nationally representative surveys [8, 17]. Subsequently, we conducted a preliminary survey round where we administered 50 selected disease conditions for scoring. Succeeding the preliminary scoring, we analysed and presented the scores followed by moderator guided discussion with the expert panel. This helped ensure in having a common understanding on the aim and approach of the Delphi exercise among the expert panel. Afterwards, we conducted the second session of the scoring exercise where experts provided scores for all the 253 disease conditions/groups in five rounds using an online Google survey sheet platform [18].

## Results

**Fig. 1:**
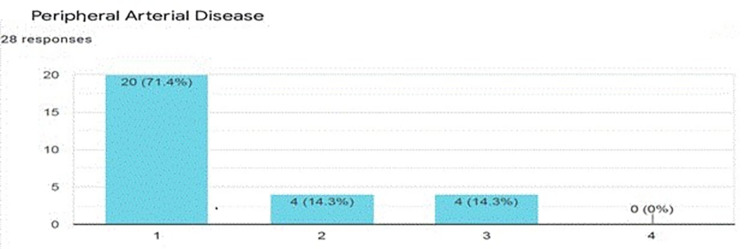
Equity score for peripheral arterial disease (a score of 1 means the condition predominantly affect urban residents and the wealthy, while a score of 4 is when the condition predominantly affect rural residents and the poor)

**Fig. 2:**
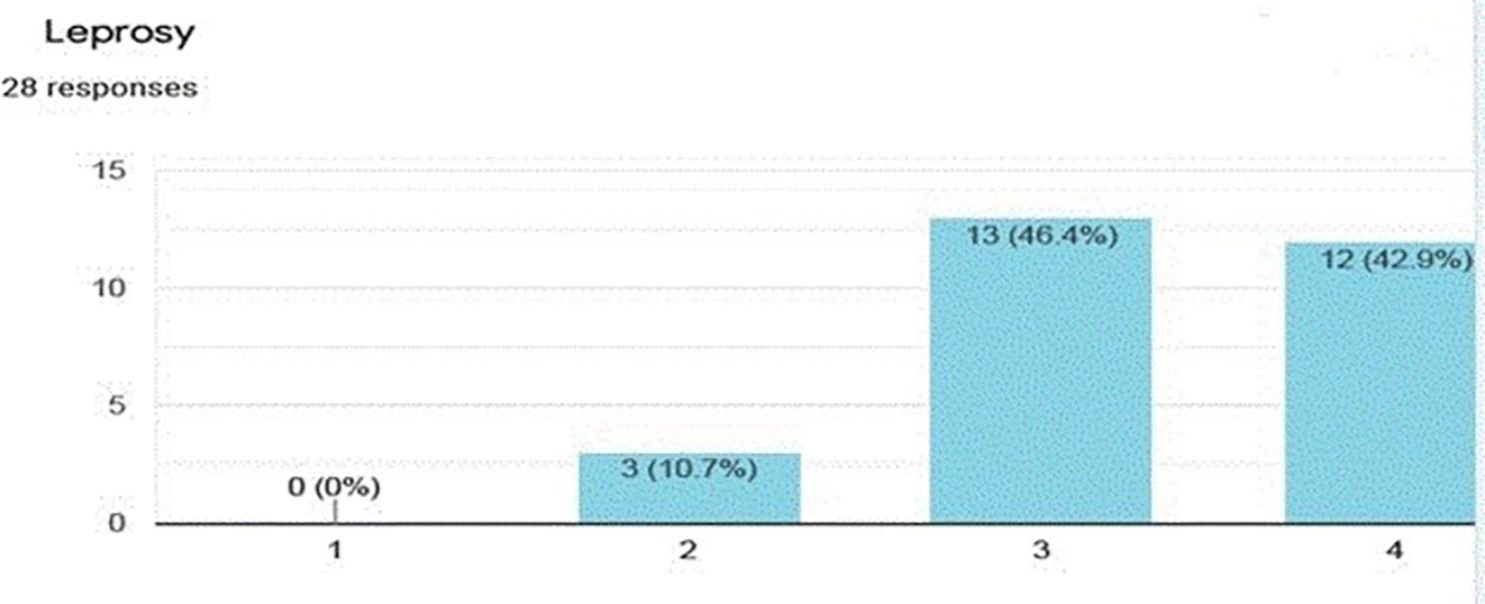
Equity score for Leprosy (a score of 1 means the condition predominantly affect urban residents and the wealthy, while a score of 4 is when the condition predominantly affect rural residents and the poor)

The average scores ranged between 1.11 for vitiligo (an acquired disorder of skin pigmentation) and 3.79 for obstetrics fistula in women (abnormal communications between the genital tract and the urinary tract that occur as the result of obstetric trauma, typically from prolonged obstructed labor). The disease conditions and their average equity score are presented in additional file II, “Expert score0” worksheet. Figures 1 and 2 (included as an example) show what the expert panel scores for two disease conditions, peripheral arteria disease and leprosy, respectively.

**Fig. 3:**
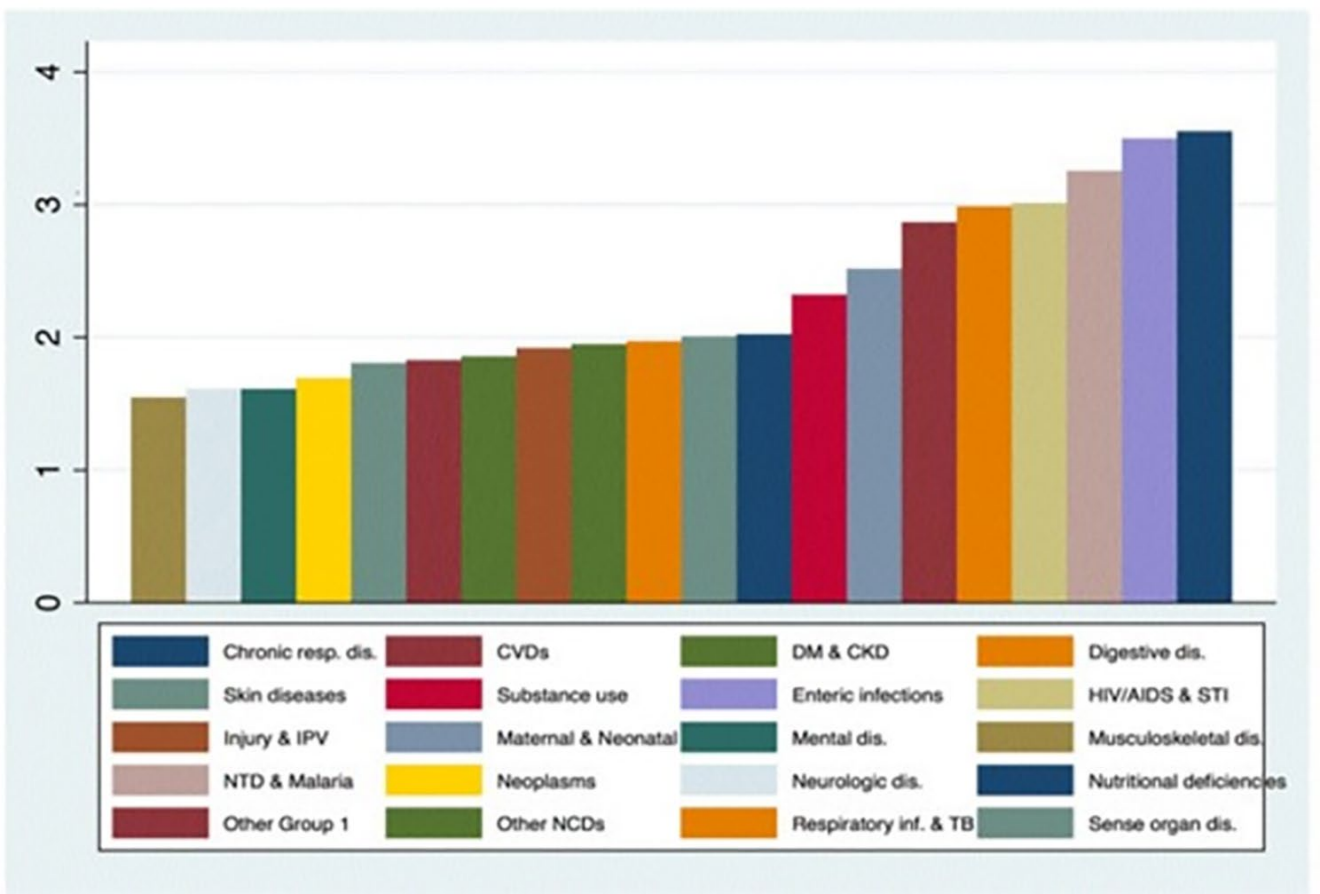
Average equity scores by GBD-level 2 disease categories

The scoring for the 253 disease conditions were based on GBD-Level 4 categorization and we aggregated them into 20 GBD-level 2 categories, the result of which is presented in Fig. 3 below. The aggregation demonstrated that nutritional deficiencies, enteric infections, neglected tropical diseases and HIV/AIDS and sexually transmitted infections received an average score of greater than three with the highest score assigned to nutritional deficiencies. Conditions such as neurologic and musculoskeletal disorders were assigned the lowest scores.

In the Delphi method we scored disease condition using wealth (poor vs. rich) and area of residence (urban vs. rural) as the equity attributes. To account for gender (for conditions affecting women) and age (conditions predominantly affecting children) that were identified as important additional attributes by the core team, we proceeded as follows: (i) we arranged the disease conditions by their scores in a descending order and categorized them into three groups (high, medium and low), presented on additional file II, “Expert score0” worksheet; (ii) we identified conditions/diseases affecting women and children including their categorization into the three categories based on average expert panel scores (in the high, medium or low). Most of the conditions affecting women and children were categorized in the high score group by the expert panel (additional file II, “MCH” worksheet); (iii) third, those maternal and childhood conditions that were in the medium or low score categories (22 conditions) were assigned the value of the disease condition with the least score from the high category group (an average score of 2.25). The rationale for this decision was as follows [9, 10, 14]: (i) the most important drivers of inequity are SES and area of residence that were taken into account in the assignment of score by the expert panel for the prevailing disease conditions in Ethiopia including those conditions that are particularly prevalent in women and children; (ii) women and children are more likely to be marginalized regarding access to health care services; and (iii) the need to account for such conditions but not higher than SES and area of residence. The expert panel decided that these additional attributes should not carry equal weight to income and area of residence and therefore decided to assign the least score from the high category group (this would allow maternal and childhood conditions to have a relatively better equity scores as compared to those conditions in the medium and low categories). The equity scores for conditions affecting women and children are presented in additional file II, “MCH” worksheet and for all conditions after accounting for these conditions is presented in additional file II, “Expert-MCH score” worksheet.

**Fig. 4:**
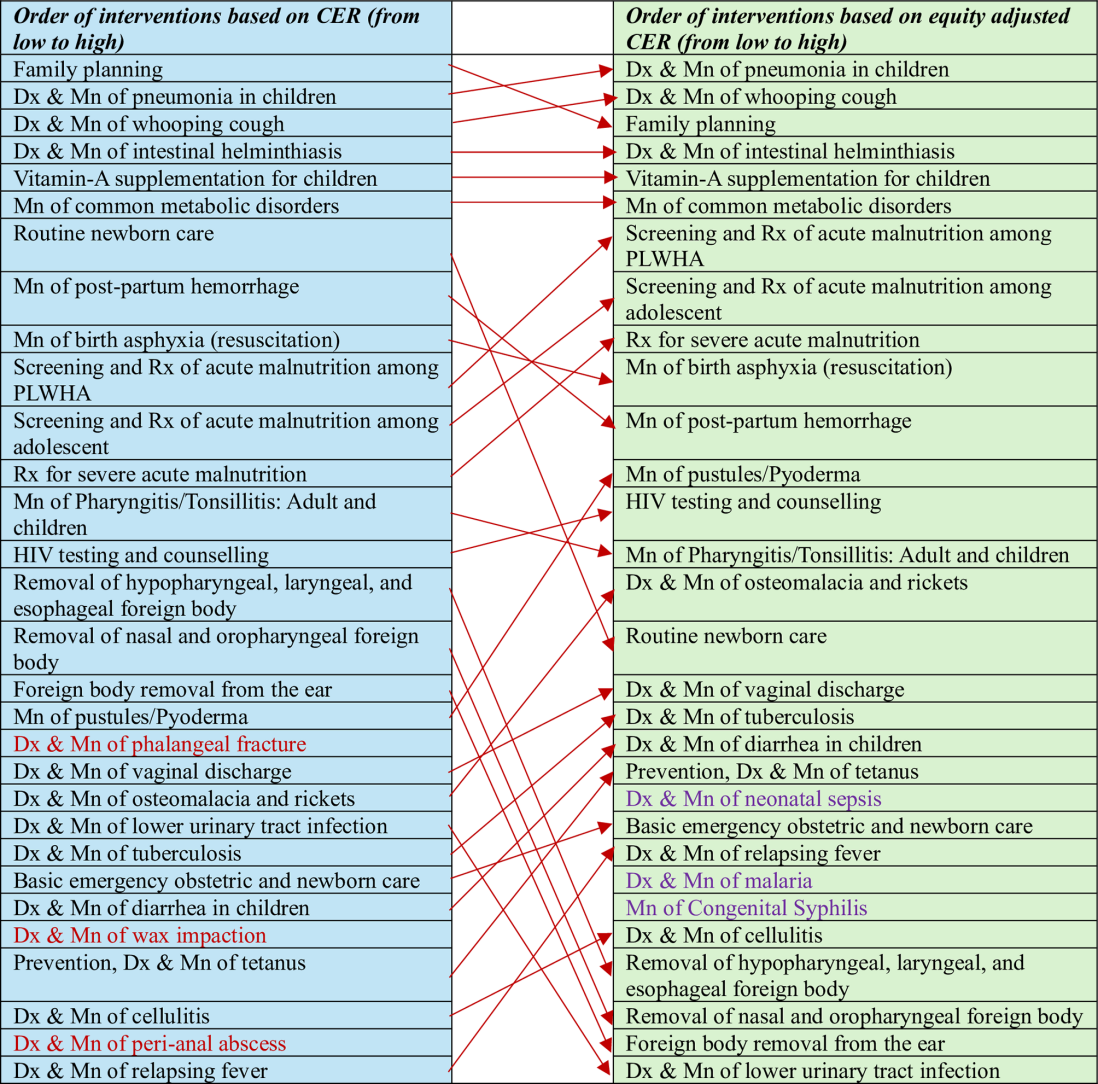
The top 30 interventions ordered based on cost-effectiveness ratios (CER) and equity adjusted CER

Once the equity scores for all the conditions based on the four equity criteria are finalized, we standardized the scores to a range between 1 and 2, corresponding to the lowest and the highest equity scores, respectively (additional file II, “Standardized score” worksheet). This is based on the expert panel consensus that health gains to the poor and rural residents should carry twice the weight of health gains accrued to the rich and urban residents. Subsequently, the scores for the disease conditions were mapped into their corresponding interventions (additional file II, “Equity scores_interventions” worksheet) along with their cost-effectiveness ratios (CER) (additional file II, “CEA-Equity” worksheet). The equity scores modify the CER, where a score of 2 will have twice the effect in modifying the CER as compared to an equity score of 1 (the CER decreases by half for interventions with an equity score of 2 but no change if an intervention’s equity score is 1), see additional file II, “Equity adjusted CEA” worksheet. The equity adjusted CEAs have resulted in a different ranking of health interventions in the league table. Figure 4 also shows the ranking and reranking of selected top 30 interventions arranged in ascending order (from lowest to highest) based on CEA and equity adjusted CEA values. The interventions in red color are no more included among the list when the interventions are prioritized using equity adjusted CEA values while those interventions in purple color are included.

## Discussion and conclusions

Health equity plays a central part in social justice. Health is not only the most important condition of human life but also a highly valued constituent of human capabilities [19]. Health is also a unique resource for realizing other objectives in life, such as better education and employment. Inequalities in health could compromise the most basic freedoms and life opportunities of individuals. Therefore, inequality in health may result in inequalities in people’s capability to function and in a denial of equality of opportunity [20].

Fairness and equity are crucial in public policy decisions and have a focal role in the context of universal health coverage (UHC) [21, 22]. Fairness and equity are important goals on the path towards progressive realization of UHC. This entails that, when countries expand the number of interventions covered, include more people, and reduce out-of-pocket payments, they must seek to do so fairly and equitably [3]. Furthermore, Alan Williams notes that “The best way to integrate efficiency and equity considerations in the provision of health care would be to attach equity weights to quality adjusted life-years (QALYs)” [23].

Similarly, in the HIBP implementation, larger equity weight was assigned to interventions that are more likely to benefit the socioeconomically marginalized and rural residents. The result has shown that the so called ‘diseases of poverty’ such as nutritional deficiencies, enteric infections, neglected tropical diseases and HIV/AIDS and sexually transmitted infections received an average score of greater than three with the highest score assigned to nutritional deficiencies [24]. Conditions such as neurologic and musculoskeletal disorders were assigned the lowest scores. An important thing to note that has been observed in this exercise was that most interventions addressing nutritional and communicable conditions were also highly cost-effective.

As compared to the unadjusted CEA’s, the equity adjusted CEA has resulted in a different ranking of health interventions in the league table influencing the probability for inclusion in the Benefits package. Various criteria are used by countries to select and implement priority interventions. A key criterion for setting health care priorities that has been usually utilized in international and national guideline is the cost-effectiveness of an interventions [25, 26]. Cost effectiveness analysis (CEA) identifies interventions that result in better health benefits relative to cost. Priority setting based solely on CEA may buy more health for available resources but will not address the other health system objectives such as equity in health and financial risk protection [3]. Besides, the public may not view prioritization of health care interventions based solely on cost-effectiveness as fair health care distribution [25]. Therefore, decision-makers should carefully consider equity criteria alongside results of cost-effectiveness analyses in the decision to fund one intervention and refuse to fund another.

Our exercise is not without limitations. We used expert panel to score disease conditions that was ultimately used to generate equity scores for interventions due to lack of disaggregated data on either access to intervention or distribution of disease burden across socioeconomic gradient or by area of residence. We presented disaggregated disease burden data by wealth and geographic location for a few conditions as an example to demonstrate the scoring process which may bias the scoring for these conditions. Additionally, some interventions could get a high equity score because they are more visible (due to donor involvement, physical manifestation of the condition or high prevalence) that might bias our findings. In the current exercise, the views of the general population were not included and the trade-off between total health gains and inequality reduction was not explicit. Furthermore, we couldn’t follow the typical Delphi iteration and controlled feedback process that continues until a predestined stopping point is reached such as consensus and stability of results mainly to reduce dominance and group conformity that may bias the result.

Disregard to distributional aspects related to health outcome across society are important limitations of health care decisions that only consider CEA. However, equity is an important criteria and studies in several countries imply that people are willing to trade off health maximization in order to prioritize the worse off and underserved populations [27]. The HIBP implementation in Ethiopia has integrated equity criterion to include interventions in the package besides the interventions cost-effectiveness. Several countries such as Sweden, Denmark, Norway and the Netherlands have explicitly included equity concerns in their health care priority setting efforts where the following key attributes were considered: severity of disease, necessity (need), social and geographic location [25, 28]. Therefore, assessing and integrating equity consideration and putting an additional weight to health benefits that accrue to the worse off individuals should be an integral part of health care priority setting.

## Electronic supplementary material

Below is the link to the electronic supplementary material.

Supplementary Material 1

Supplementary Material 2

Supplementary Material 3

## Data Availability

The data supporting the results of the study are provided within the manuscript and supplementary files.

## Footnotes

1 The HBP for CBHI included treatment services for conditions that have high prevalence and low costs plus treatment for the following services without a specific evaluation criterion: surgical management, benign prostate hypertrophy, laparotomy, hysterectomy, appendectomy, etc. It also includes burn injuries (2nd degree and beyond), treatment of diabetes mellitus, hypertension, fractures, and caesarian sections.
