# Supplementary material for: Equity considerations for the implementation of health insurance benefit package in Ethiopia: result of expert Delphi exercise
Source: Int J Equity Health. 2024 Sep 11;23:182. doi: 10.1186/s12939-024-02226-z (PMC11389339; doi:10.1186/s12939-024-02226-z)
Supplement: Supplementary file 1 — Supplementary Material 1 [file 12939_2024_2226_MOESM1_ESM.docx]

Additional file I: Framework for equity score

13-Apr-2022

Dear Experts [ Name ]:

We would like to appreciate your participation in this Equity Delphi process representing your organization from universities, public health institutes, medical associations, patient groups, health facilities, patterns, and the Ministry of Health of Ethiopia.

Equity as a criterion arises from the policy commitment of the government and local social values to create a fair and just health status for the different population groups in Ethiopia. In the redesign of the Ethiopian Health Insurance Benefits package, the equity criterion will be applied in a way that gives higher scores for diseases which mainly affect the worse off. Disease condition which does not specifically affect the worst off will be given lower equity scores.

The worse off in the Ethiopian context are defined as being children less than five years of age, women in the reproductive age group, the economically poor, and populations who live in rural areas.

We have captured equity scores for age and gender dimensions through different data sources. Therefore, in this workshop, we will evaluate the equity scores for diseases through their impact on the economically poor and the rural (both pastoralist and agrarian) population.

Instruction to panel

Today, using the Delphi technique, a panel of experts--you--will score disease from 1 to 4; where 4 indicates disease/diagnosis which is dominant in the worse-off, and 1 indicates disease/diagnosis which is not dominant in the worse off/ which do not specifically affect the worst off. While rates 2 and 3 are grades that could be assigned based on the extent/degree that a disease impacts the worst off. When scoring the disease conditions, please try to answer the following question:

To what extent would “Disease x” affect the worse off (i.e., the economically poor and rural population)?”

Please score the disease/diagnosis condition on a scale from 1 to 4, where 4 = high equity score, and 1 = low equity score.

For example, if you believe that "X" disease is disproportionally concentrated among the worse off groups, you will give it a high equity score. If you believe that "Y" disease is equally distributed among the worse off and the better off or more prevalent among the better off, you will give it a low equity score.
