# Supplementary material for: Equity considerations for the implementation of health insurance benefit package in Ethiopia: result of expert Delphi exercise
Source: Int J Equity Health. 2024 Sep 11;23:182. doi: 10.1186/s12939-024-02226-z (PMC11389339; doi:10.1186/s12939-024-02226-z)
Supplement: Supplementary file 3 — Supplementary Material 3 [file 12939_2024_2226_MOESM3_ESM.docx]

**Annex III**

**Health Insurance Benefit Package: workshop on equity, participants list**

|  | *Expertise* | *Institution* |
| --- | --- | --- |
| 1 | Pediatrician | Ministry of Health, Maternal and Child health directorate |
| 2 | Reproductive health expert | World Health Organization |
| 3 | Pediatrician | Ministry of Health, Health extension program |
| 4 | General Practitioner | Ministry of Health, Health extension program |
| 5 | Emergency specialist | MoH Emergency |
| 6 | Dermatologist | Armeur Hansen Research Institute |
| 7 | Representative, Cancer society | Consortium of Ethiopian Non-Communicable Diseases Association (CENCDA) |
| 8 | Representative, Kidney society | Consortium of Ethiopian Non-Communicable Diseases Association (CENCDA) |
| 9 | Patient representative (chief executive officer) | CENCDA |
| 10 | Epidemiologist | Addis Ababa University |
| 11 | Internist | St. Paul Hospital Millennium Medical College |
| 12 | Health economist | Addis Ababa University |
| 13 | General practitioner | Women’s physician association |
| 14 | Surgeon | Adama general hospital |
| 15 | Orthopedician | Adama general hospital |
| 16 | Nurse | Beletshachew health center |
| 17 | Internist and NCD expert | Center for Integration Sciences |
| 18 | Psychiatrist | Amanuel mental specialized hospital |
| 19 | Neurologist | Tikur Anbessa Specialized Hospital |
| 20 | Family medicine | Tikur Anbessa Specialized Hospital |
| 21 | Public health expert | Ethiopian Public Health Institute |
| 22 | Researcher | International Institute of Primary Healthcare |
| 23 | Public health expert | Ethiopian Medical Association |
| 24 | Medical director | Primary Hospital (Afar) |
| 25 | Medical director | Primary Hospital region (Debresina) |
| 26 | Medical director | Jigjiga hospital |
| 27 | General practitioner | Women’s physician association |
| 28 | Representative | Women’s association |
